# Personal PM_2.5_ Exposure Monitoring of Informal Cooking Vendors at Indoor and Outdoor Markets in Johannesburg, South Africa

**DOI:** 10.3390/ijerph20032465

**Published:** 2023-01-30

**Authors:** Maasago Mercy Sepadi, Vusumuzi Nkosi

**Affiliations:** 1 Department of Environmental Health, Faculty of Health Sciences, Doornfontein Campus, University of Johannesburg, Johannesburg 2094, South Africa; 2 Environment and Health Research Unit, South African Medical Research Council, Johannesburg 2094, South Africa; 3 School of Health Systems and Public Health, Faculty of Health Sciences, University of Pretoria, Pretoria 0001, South Africa

**Keywords:** air pollution, particulate matter, street vendors, exposure assessment, occupational exposure, environmental health

## Abstract

Air pollutants of concern include particulate matter (PM) in fine size fractions. Thus far, a few studies have been conducted to study the adverse health effects of environmental and occupational air pollutants among informal vendors in big cities in South Africa. Informal vendors in these cities may experience higher exposure to road dust, cooking fumes, and air pollution. This exposure assessment was part of a health risk assessment study of vendors. The objective of this exposure assessment was to determine the differences between outdoor and indoor informal vendors’ personal PM_2.5_ exposures during trading hours. A walkthrough survey was conducted to map the homogeneous exposure groups (HEGs) at vendor markets for sampling purposes, and one market was selected from each of the three identified HEGs. Twenty-five informal cooked food vendors from both indoor (inside buildings) and outdoor (street or roadside vendors) markets in the inner city of Johannesburg, South Africa, participated in the study. HEG-1 were vendors from indoor stalls who used electricity and gas for cooking (10 vendors), HEG-2 was composed of informal outdoor vendors at a fenced site market who used open fire for cooking (10 vendors), and HEG-3 (5 vendors) were roadside vendors who used gas for cooking. Cooking vendors from outdoor markets recorded higher TWA concentrations than indoor market vendors. The vendors’ PM_2.5_ concentrations ranged from <0.01 mg/m^3^ to 0.77 mg/m^3^. The mean concentrations of PM_2.5_ were found to be 0.12 mg/m^3^, and 0.18 mg/m^3^ for HEG-2, and HEG-3, respectively. HEG-2 recorded the highest PM_2.5_ TWA concentrations, followed by HEG-3 and HEG-1. All concentrations were below the South African occupational exposure limit. The findings point to the need for further research into the health risks associated with outdoor cooking vendors, particularly those who utilize open fires.

## 1. Introduction

According to World Health Organization (WHO) estimates, long-term exposure to ambient fine particle air pollution (PM_2.5_) resulted in 102 million lost years of healthy living and 4 million deaths in 2015, accounting for 7.6% of all deaths worldwide, ranking it as the fifth most significant global risk factor that year [1]. Particulate matter, defined as particles with a diameter of less than 2.5 μm (PM_2.5_), is regarded as the most dangerous to human health due to its microscopic size [2,3]. PM is the name for an airborne mixture of liquid droplets and solid particles, such as dust, dirt, soot, or smoke [4]. Although there are many other sources of air pollution, incomplete combustion processes in motor vehicles, industrial facilities, power plants, and kitchen activities are the main culprits [2]. 

According to pollution measurements from satellites, researchers have determined that the air in Johannesburg, South Africa, is the worst in the nation and that it shortens people’s lives by 3.23 years [2]. Currently, Johannesburg’s PM_2.5_ concentrations are 1.1 times the WHO annual air quality guideline value [2].

The environment makes a major contribution to public survival in terms of health, and this includes places of work. According to epidemiological research, there is a connection between PM_2.5_ in the indoor and outdoor environment and diseases that are significant for public health, such as malignancies, respiratory illnesses, and cardiovascular diseases such as stroke and ischemic heart disease [5]. There are two different categories of informal vendors, each with their own set of difficulties. Informal vendors frequently operate from outdoor or street locations without a formal building structure, including tents, cartons, or mobile food trailers. These vendors conduct their business in public areas close to downtown streets, marketplaces, or parks (such as a storefront, a sidewalk, a roadside, or pavement) [6]. Some informal vendors, however, operate markets in discrete locations within an enclosed permanent brick structure. These vendors may operate in semi-public spaces, such as inside market buildings and public transportation stations [6], as well as train stations, taxi ranks, and other public places. The burden of disease associated with environmental exposures disproportionately impacts outdoor vendors in big cities all over the world.

This exposure assessment is part of a respiratory health risk and impact assessment Ph.D. study conducted in the inner city of Johannesburg [7,8]. According to the authors’ knowledge, there has never been a study that examined the dangers to street vendors’ respiratory health due to personal PM mass concentration in South Africa. Moreover, the assessment seeks to compare indoor (inside a building) vendors and outdoor (roadside or street) vendor markets’ PM_2.5_ exposures. 

## 2. Materials and Methods

This cross-sectional study was carried out in the inner city of Johannesburg in South Africa [7] at selected indoor (within buildings) and outdoor (on the side of the road or in the street) markets; this region was identified as the informal trade hub region within the City of Johannesburg Municipality [7]. This PM_2.5_ air pollution sampling took place during the winter season. The winter or cold winter season has been observed to increase pollution due to an increase in PM and CO pollutants from activities such as wood burning [9,10]. Furthermore, in comparison to summer or hot weather, air pollution is present on the ground for longer periods of time during the winter, resulting in a higher or longer exposure rate [9,10].

When determining the sample size of the vendors for this exposure assessment, the NIOSH occupational exposure sampling strategy manual (OESSM) was used [11]. The results of the walkthrough survey, on the other hand, led to the division of the vendor markets into three homogeneous exposure groups (HEG) based on the activities and locations of the markets, as well as the cooking fuels used there. This sampling method obtained a high probability of sampling a high-risk vendor while minimizing the sample load. From each HEG, sample size calculations for a maximum risk subgroup were made. The sample size was determined for the top 20% (r = 0.20) at a confidence level of 0.95 (=0.05) [11]. There were 25 and 26 cooking vendors at the HEG-1 (inside market with electricity and gas stove usage) and HEG-2 (outdoor market with open fire usage) selected locations, respectively, and 10 of those vendors provided the sample size for each HEG group’s personal air sampling as per the OESSM required number of measured employees. Then, for HEG-3 (an outdoor market with gas stove usage), it was discovered that there were 9 stationed cooking vendors stalls, however on the day of sampling, only 7 stalls were functional, so 5 of the vendors made the sample size required by the NIOSH manual. 

A total of 25 informal cooking vendors from the selected HEG in Johannesburg’s inner city took part in the study to carry out this exposure evaluation. This sampling approach examined each participant’s occupational exposure to PM_2.5_ pollutants during the peak work hours of the vendor’s work activities. In order to cover the informal vendors’ busiest operating hours throughout a single work shift, the sampling program was carried out during the week (Monday to Friday) between 8 am and 3 pm [7,8,12] as the majority of the informal vendors operate between 5 am and 6 pm. Due to financial restrictions, the individual vendor PM_2.5_ samples were obtained as part of a two-work-day program under the worst possible condition [6], which was on 30 June 2022 and on 24 August 2022 in the wintertime. The personal air sampling was conducted between 9 am and 4 pm on the sampling date.

**Figure 1:**
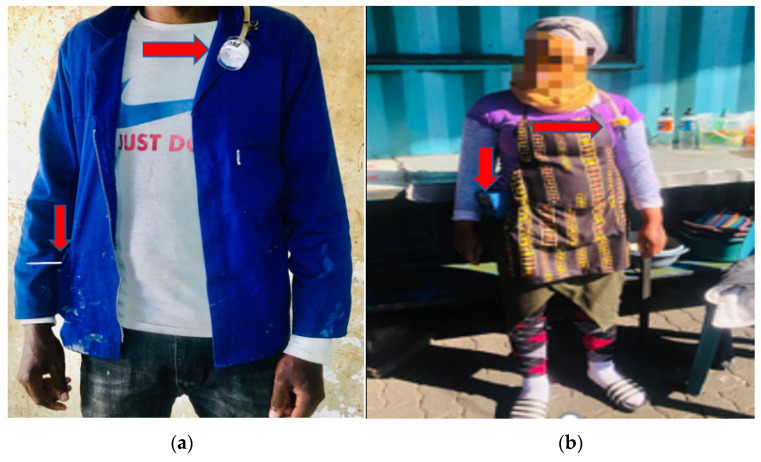
(**a**) An indoor informal cooking vendor wearing a sampling device. (**b**) An outdoor informal cooking vendor wearing a sampling device.

Personal air sampling is an important method of assessing occupational exposure to dust, gases and vapours. Strict adherence to the sampling strategy as prescribed in OESSM [11] was followed using the GilAir-3 personal air sampling pump. This assessment was conducted by attaching the sampling device to the employee’s jacket collar within the breathing zone (Figure 1). Figure 1a,b shows the positioning of the device on the informal vendors, as shown with arrows. Each vendor used their own sampling equipment once, and calibration of the sample was conducted before and after usage. Care was taken to prevent the equipment from interfering with the employee’s movements in the area, and the employee proceeded with their normal duties.

The information that identifies each sampler was recorded on a sampling field sheet, reflecting information such as the sampler number, sampling area, sampling method, duration and environmental conditions [7]. All personal air sampling pumps were properly calibrated before sampling commenced at 2.2 L/min and again on completion of the survey. The samples were transported to a SANAS 17,025 accredited laboratory in a secure container. Gravimetric analysis of samples followed MDHS 14/4 methodology [13]. All TWA concentrations were compared to the occupational exposure limits (OEL) of 5 mg/m^3^, as per the Regulations for Hazardous Chemical Agents, 2021, promulgated under the Occupational Health and Safety Act 85 of 1993 [14]. SPSS version 28 [15] was used for descriptive frequencies, and Microsoft Excel 2016 was used for calculations [16].

## 3. Results

### 3.1. Identifying Homogeneous Exposure Groups

Following the walkthrough survey, the vendor marketplaces were divided into three homogenous exposure groups (HEG) based on the locations, activities undertaken and cooking fuel used (Table S1) in order to map the occupational risks (Table S1). According to the WHO (2022), 2.4 billion people worldwide (or almost a third of the world’s population) cook over open flames or inefficient stoves that burn coal, kerosene, biomass (wood, animal dung, and crop waste), and a combination of these fuels [17]. For informal cooking vendors, this may contribute to dangerous levels of indoor and outdoor air pollution. Of the 16 markets, 44% (n = 7) were markets in HEG-1, which are indoor or inside building markets that mostly use electricity and gas. The second group (HEG-2) represented 31% (n = 5) of the outdoor vendor markets, which mostly use open fire (firewood and charcoal) and are exposed to traffic, and the third group (HEG-3) represented 25% (n = 4) of the outdoor vendors, which are located on roadsides and sidewalks and mostly use gas. The selected markets were identified as Market 1 (HEG-1), Market 2 (HEG-3) and Market 3 (HEG-2), as per Table S1. In terms of location, Markets 1 (HEG-1) and 2 (HEG-3) were closer to each other and far apart from Market 3 (HEG-2). HEG-1 was 4.7 km away from HEG-2 and 700 m away from HEG-3 were 4.5 km apart [18]. 

#### Description of Selected Markets for the Air Sampling Program

**Figure 2:**
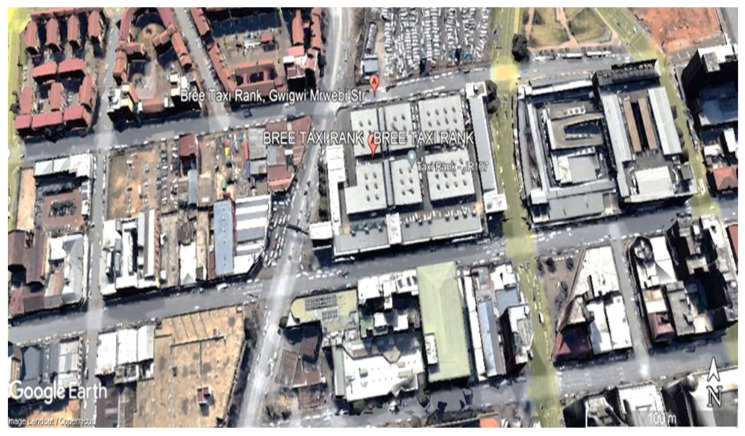
A map showing sampling location (HEG-1 market) in the inner city of Johannesburg [18].

Market 1 in Table S1 is characterized as a particular indoor market taken from HEG-1. This transportation station houses the market. Shops, apartments, a park, local traffic, and a highway are all close to the market (Figure 2). Cooking vendors are accommodated in fully constructed booths with cemented floors, brick walls, and metal roofs, in addition to other types of vendors (Table S1). There are windows and an opening to the outdoors in the stall seating area, providing natural ventilation, but there are no windows in the individual stall rooms.

**Figure 3:**
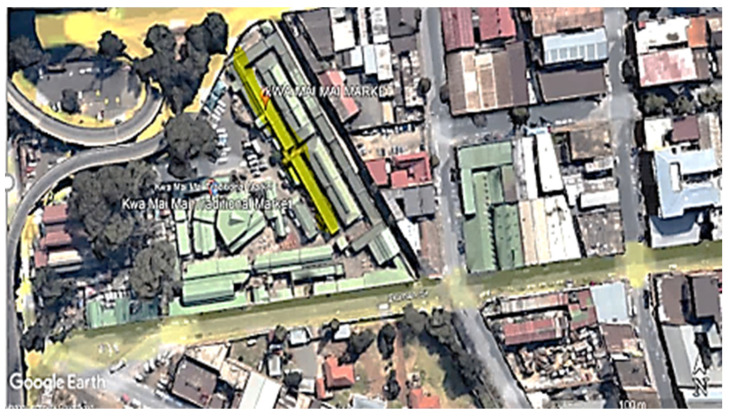
A map showing sampling location (HEG-2 market) in the inner city of Johannesburg [18].

From HEG-2, Market 3 in Table S1 is described as an outdoor market. This outdoor market is fenced with a gate entrance to the stall area. The vendors are not in proper brick-building structures. There are fully built steel stalls used as storage and open outdoor spaces used as cooking areas. The cooking area is only paved, with no roof or side structures (Table S1). The market is surrounded by shops, residential flats and local traffic and is also surrounded by industrial premises and offensive trades, which are businesses with activities such as recycling and car fixing (Figure 3). The vendors spent more time in the cooking area exposed to open fire.

**Figure 4:**
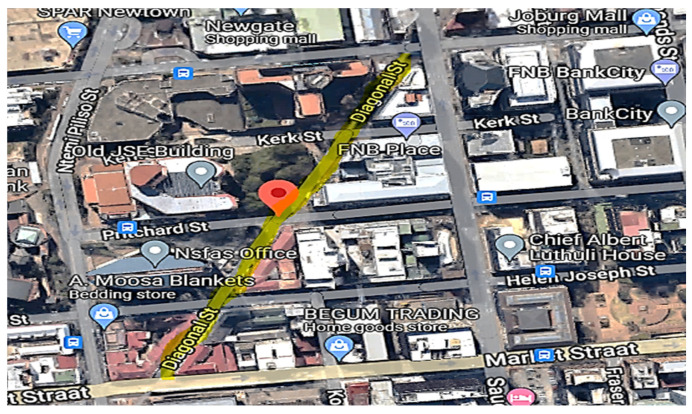
A map showing sampling location (HEG-3 market) in the inner city of Johannesburg [18].

From HEG-3, Market 2 in Table S1 is described as an outdoor market. This sidewalk market is in between shops. The market is surrounded by shops, residential flats and local traffic (Figure 4). These vendors who use gas for cooking purposes are not in proper building structures but inside steel-made stalls with cemented or paved floors and metal roofs (Table S1). The stalls are not fully covered on the sides, allowing for natural ventilation.

### 3.2. PM_2.5_ Personal Vendor Exposures in Indoor and Outdoor Market

Table 1 and Table S2 present the PM_2.5_ personal exposure measurements at selected informal vendor markets in the inner city of Johannesburg. There were a total of 25 personal air samples collected; (n = 10; 40%) from HEG-1 (indoor) and HEG-2 (outdoor), respectively, and (n = 5; 20%) from HEG-3 (outdoor) (Table 1). The TWA concentrations ranged from <0.01 mg/m^3^ to 0.77 mg/m^3^. The lowest personal exposures were at a TWA concentration of <0.01 mg/m^3^ (n = 5; 20%), which was mostly seen in HEG-1. A TWA concentration of 0.01 mg/m^3^ (n = 4; 16%) was the second most frequent value, followed by 0.16 mg/m^3^ (n = 3; 12%). Comparing HEG-2 to HEG-1 and HEG-3, the personal vendor exposures were the highest in HEG-2.

The TWA concentrations of vendors who worked indoors (HEG-1) varied from <0.01 mg/m^3^ to 0.77 mg/m^3^ (Table 1). The observed PM_2.5_ TWA concentrations were as follows: three indoor market vendor’s personal exposures were less than 0.01 mg/m^3^; two were exposed to 0.01 mg/m^3^, and the remaining five indoor market vendors displayed various PM_2.5_ values, 0.02 mg/m^3^, 0.04 mg/m^3^, 0.09 mg/m^3^, 0.24 mg/m^3^, and 0.77 mg/m^3^. For HEG-2, the concentration of PM_2.5_ varied, with only a single concentration of <0.01 mg/m^3^ and the highest peaking at 0.48 mg/m^3^. The other measured PM_2.5_ TWA concentrations were found to be 0.01 mg/m^3^, 0.11 mg/m^3^ for two of the vendors, 0.16 mg/m^3^ for another two vendors, 0.41 mg/m^3^, 0.17 mg/m^3^, and 0.18 mg/m^3^. The HEG-3 concentrations of PM_2.5_ varied, with the lowest value being <0.01 mg/m^3^ and the maximum value being 0.62 mg/m^3^ (Table 1 and Table S1). PM_2.5_ TWA values were 0.01 mg/m^3^, 0.12 mg/m^3^, 0.16 mg/m^3^ and 0.62 mg/m^3^, and, similar to HEG-2, there was only a single personal exposure of <0.01 mg/m^3^. The fine particulate matter concentrations in all homogeneous exposure groups were far below the South African OEL [12] (Table S2). Even though all concentrations complied with respective OEL, approximately three values (0.77 mg/m^3^ in HEG-1; 0.48 mg/m^3^ and 0.62 mg/m^3^ in HEG-3) were above 10% (0.5 mg/m^3^) of the OEL. 

#### 3.2.1. Average Indoor and Outdoor Vendor Homogeneous Exposure Groups Exposures

The majority of the informal cooking vendors from outdoor markets recorded higher TWA concentrations exposures than vendors from indoor markets (Table 2 and Table S2). The vendors’ mean concentrations of PM_2.5_ were found to be 0.12 mg/m^3^, 0.18 mg/m^3^, and 0.18 mg/m^3^ at HEG-1, HEG-2, and HEG-3, respectively. There was some similarity in the mean concentrations of the outdoor vendor groups (HEG-2 and HEG-3), which were higher than the indoor group with 0.07 mg/m^3^.

Despite the lack of research on vendors’ personal air exposure assessment, the higher personal exposures in HEG-2 and HEG-3 compared to indoor market (HEG-1) exposures were not entirely unexpected. The expectation was due to recent vendor studies that reported on ambient air exposure, such as a study in Malaysia which found higher average PM_2.5_ exposures among roadside areas (31.05 μg/m^3^), as compared to the non-exposed reference group (19.41 μg/m^3^) [12]; another similar study was conducted in Bangkok, in which PM_2.5_ concentrations were also much higher along the road than in the residential area [19].

#### 3.2.2. Correlations between the TWA PM_2.5_ Concentrations and Meteorological Information

In order to comprehend PM_2.5_ variability, it is crucial to quantify interactions between meteorological conditions and PM_2.5_ concentrations. There were three meteorological factors, including temperature, humidity, rainfall and wind speed, for the two-day sampling program that took place on Thursday, 30 June 2022 and Wednesday, 24 August 2022. The weather conditions in Johannesburg on 30 June 2022 were sunny. The morning forecast (06:00–12:00) recorded the humidity (54%), temperature (min. 7 °C and max. 13 °C), and wind speed (9 km/h, SSW); and in the afternoon (12:00–18:00), it recorded the humidity (18%), temperature (min. 14 °C and max. 16 °C), and wind speed (14 km/h, E). Wednesday, 24 August 2022 was a sunny day; the morning forecast (06:00–12:00) recorded the humidity (80%), temperature (min. 8 °C and max. 8 °C), and wind speed (17 km/h, NNE); in the afternoon (12:00–18:00), it recorded the humidity (34%), temperature (min. 19°C and max. 19 °C), and wind speed (7 km/h, E). Rainfall was 0 mm on both dates [20,21]. All participants of the HEG-3 sampling program were instituted on 30 June 2022. The HEG-1’s sampling program was conducted on 30 June 2022 for participants 1 to 5, and the sampling program for participants 5 to 10 was conducted on 24 August 2022 (Table S2). All HEG-2 sampling points were collected on 24 August 2022.

As per the recorded meteorological conditions, winter has generally been found to have lower humidity levels when compared to summer and other seasons with extremely high humidity, ranging from 80% to 100%. Therefore, it is a harmful season as the particles in winter are lighter, remaining in the air due to lower humidity, as opposed to summer when they are too heavy to remain in the air and result in dry depositions, where particles land on the ground [2,3,7]. Temperature and PM_2.5_ have a significant positive association due to their effect on the formation of particles. Consequently, the photochemical reaction between precursors can be aided by high temperatures [3,22]. The influence of precipitation on pollutant concentration is that, through wet deposition, precipitation can significantly lower PM_2.5_ mass concentrations [22]. PM readings are also influenced by wind direction and speed, as higher PM values are observed with lower wind speeds [3,9,10,22]. Low wind speeds can blow away pollutants within a specific geographic range, whereas high wind speeds can carry large amounts of pollutants from a great distance [22]. For instance, in the study by Wang et al. (2015), the value of outdoor PM_2.5_ mass concentrations attained a moderate pollution level in the spring and winter, when the outdoor wind speed was less than 1 m/s; on the other hand, the indoor PM_2.5_ mass concentrations in winter exceeded the air quality requirement [22]. However, wind speed and relative humidity exhibited a moderate-to-weak negative connection with PM concentration, while average temperature had a substantial negative correlation [23].

This factor may have impacted the number of higher PM_2.5_ concentrations in HEG-2 and HEG-1 of the samples collected on 24 August 2022, where the temperature was higher as compared to the temperature of 30 June 2022 in the same location. The 5 samples in HEG-1 collected on 24 August 2022 had TWA concentrations of 0.01 mg/m^3^ to 0.77 mg/m^3^, higher than the 5 samples collected on 30 June 2022, which were between <0.01 mg/m^3^ and 0.04 mg/m^3^, with three of the samples recording a concentration of <0.01 mg/m^3^ (Table S2). The highest PM_2.5_ TWA concentration was recorded at 0.77 mg/m^3^ in HEG-1; other than the meteorological conditions, the vendor sampling field sheet notes reflect that the vendor was also using a gas stove for cooking on that day, which could have contributed to the said exposure. However, comparing the mean TWA concentration of outdoor samples to indoor samples showed that for those collected in similar weather conditions, most outdoor concentrations were higher than indoor concentrations (Table S2).

## 4. Discussion

The PM_2.5_ exposures showed that outdoor vendors are more exposed as compared to indoor vendors. The highest personal air sample in HEG-1 (indoor market) was 0.77 mg/m^3^; this value was 0.48 mg/m^3^ for HEG-2 (outdoor market), and the highest value peaked at 0.63 mg/m^3^ for HEG-3, with no personal exposures exceeding the national OEL The homogeneous exposure groups’ mean TWA concentrations were found to be 0.18 mg/m^3^ at both HEG-2 and HEG-3 (outdoor markets), higher than HEG-1 (indoor), which was found to be 0.12 mg/m^3^ (Table 2). The bulk of research studies that looked at the relationship between vendor’s particulate matter exposure and the health impact used area or static air sampling techniques rather than personal air sampling techniques, and most of these investigations were conducted abroad. However, individual worker or personal air sampling is very important for assessing the potential hazards of inhaled particles in a workplace. The results of these studies reported higher exposures at outdoor or roadside areas [12,19]. Furthermore, these studies’ results reported the general ambient air exposure for any type of vendor, not particularly cooking vendors.

The correlation analysis between PM_2.5_ concentration and meteorological data revealed a connection with daily temperature, with the slightly higher temperature of 24 August 2022 affecting the higher concentrations at HEG-1’s 5 samples and HEG-2 as compared to the samples collected at HEG-1 and HEG-3 on 30 June 2022. In Accra’s traffic hotspots, where street vending and hawking predominate, PM_2.5_ and PM_10_ levels are high during both the wet and dry seasons as compared to indoor work settings [24].

Compared to indoor vendors, the elevated concentrations of PM_2.5_ amongst outdoor vendors (HEG-2 and HEG-3) may be due to the general environmental and occupational conditions. The higher concentrations may emanate from the type of fuel used (open fire and gas), as well as vehicle emissions and construction activities that are evident in the inner city of Johannesburg, especially with various numbers of vehicles, both public and private, being poorly maintained. Additionally, it is stated that roads are made up of solid particles that are produced by mechanical processing, notably by the friction of car tires traveling over unpaved dirt roads and dust-covered paved roads, with increased exposure from seasonal winds and vehicle speed [18]. Various studies have stated that smoke from wood combustion and automobile emissions are two major sources of winter PM_2.5_ [7,9,12,19,24,25]

The increase in PM_2.5_ concentrations in outdoor markets may have also been impacted by the industrial and offensive trades, such as recycling and car fixing businesses, that surround the outdoor markets. The outdoor markets were discovered to be in poor sanitary conditions and to be dustier than HEG-1. The use of electric stoves, ventilation methods, and good sanitary conditions may have contributed to the majority of lower concentration values in HEG-1 (indoor market). Moreover, in Market 3 (HEG-2), most of the informal cooking vendors may be impacted by the fact that most of the vendors in this market generally cook in a standing position directly exposed to the combustion of the open fire and take longer at the open fire to ensure that the food is not burnt (Table S1). Unclean conditions in the working environments of vendors were also reported in the results of the very few studies carried out in various South African cities [8,26] and similar studies in other countries [6,8,12,27].

There is still a risk to health even though all concentrations were lower than the OEL. The present set of OELs is causing concern among various institutions, such as the Institute of Occupational Medicine (IOM). According to the IOM and NIOH, the present British occupational exposure guidelines for airborne dust are hazardous, and businesses should make an effort to lower exposures to help prevent new incidences of respiratory disease among their workers [28]. The IOM has further advised lowering the OELs to as low as 1 mg/m^3^ [28], while NIOH (2016) has suggested halving the current values to lessen health consequences [29].

The impact of exposure to PM_2.5_ on the global burden of disease continues to increase [1,30,31]. Long-term exposure to outdoor air pollution and traffic-related air pollution has been found to shorten life expectancy [31], while long-term exposure to combustion-related fine particulate air pollution has been found to be a risk factor for cardiac, pulmonary, and chronic bronchitis, as well as decreased lung capacity and lung cancer mortality [12,19,30,31,32]. High PM_2.5_ exposures among street vendors were linked to higher rates of coughing, catarrh (postnasal drip), sneezing, rapid heartbeat, irregular heartbeat, severe chest pains, fainting spells, migraines, and dizziness when compared to office employees; while the probabilities of developing dermatitis, having a rapid or irregular heartbeat, or experiencing severe chest pain rose with low and medium PM_2.5_ exposures, respectively [24]. As per a study by the WHO, exposure to fine particles in the ambient environment and indoors results in millions of premature deaths each year, making air pollution a serious hazard to environmental health [31,32]. For informal indoor vendors, while air conditioning reduces pollution, it may not be enough to prevent respiratory problems caused by the closeness of the indoor shops to traffic and automobile emissions [23].

The health effects of exposure to PM_2.5_ has an impact on the entire health system of the country. A study conducted in China found that PM_2.5_ had a significant impact on healthcare expenditure for respiratory diseases, with a positive impact on total healthcare expenditure, drug expenditure, and antibiotic expenditure [33]. Using the IQAir global air quality database, IQAir and Greenpeace Southeast Asia have created a cost calculator that determines the financial and health consequences of air pollution in some of the world’s largest cities [2]. This estimate is based on an algorithm that incorporates the following data inputs to determine costs over the course of a full calendar year: real-time data from ground-level air quality sensors managed by the IQAir air quality database, including PM_2.5_ and NO_2_ data, scientific risk models, and population and health data. In 2020, nearly 200,000 deaths and a total cost of USD 106 billion were recorded in the top 5 most populous cities in the world (Tokyo, Japan, New Delhi, India, Shanghai, China, Sao Paulo, Brazil and Mexico City, Mexico). In 2021, air pollution in Johannesburg had an estimated responsibility for 120 deaths and a total cost of USD 47,000,000 [2].

### Study Limitations

This cross-sectional study aimed at describing the present conditions of the markets and examined the amount of PM_2.5_ the vendors are exposed to on an 8-h work duration in each market, with a comparison of outdoor and indoor vendors. Our best understanding indicates that this study is the first to look into vendors’ occupational exposure to PM_2.5_ using personal air sampling techniques in South Africa; hence, there is a lack of literature to support the findings of this study.

This baseline exposure assessment was conducted on the maximum exposed groups with no control group. The number of participants followed the homogeneous exposure group sampling program by NIOSH, which sampled only the highest-risk workers in the workplace and did not include a control group. Future studies should target longitudinal study designs that can measure personal exposure over time and conduct lung examinations on the vendors.

## 5. Conclusions

In conclusion, there is a lack of studies in SA about the occupational exposure of street sellers to air pollutants such as PM_2.5_. Most studies have focused on exposure using fixed stations; however, a good indicator of occupational exposure to airborne contaminants is personal air samplers that are placed on the worker during their occupational activities. According to this study and the current international literature, PM_2.5_ has differed for those indoor or inside buildings vendors and the outdoor vendors who sell across the street [8]. The PM_2.5_ sources of concern for this study were primarily road dust, motor vehicle emissions and dirty cooking fuels [7,8]. Outdoor occupations, especially those working along the road, are characterized by prolonged exposure to high concentrations of outdoor air pollutants due to staying near roads or close to traffic lights where vehicles are required to stop and leave their engines running [12,19,25].

The roads are characterized by heavy traffic, especially during morning and afternoon peak hours, with different types of petrol- and diesel-using vehicles such as trucks, mini-buses, private cars, buses, and scooters. There is strong evidence that street vendors’ exposure to PM_2.5_ makes respiratory and cardiovascular problems more common. The emissions from roads are discharged in close proximity to human receptors, and fossil fuels, which are also used in informal vendors’ cooking activities, have been recorded to result in approximately 1.8 billion days of lost work due to illnesses connected to PM_2.5_, as well as 4.5 million premature deaths annually and USD 8 billion in daily costs [2]. The outdoor vendors are the most vulnerable group.

The general air quality index for PM_2.5_ showed a good status for Johannesburg in South Africa, showing satisfactory air quality with little or no risk to health for 24-h exposures [2]. However, as informed by the current literature, this study assumes that other factors such as age, the usage of respiratory protective equipment, and sensitive groups may experience minor to moderate symptoms from long-term or continuous exposure [2,7,8].

Therefore, in order to prevent any future health risk and costs to the health system due to PM_2.5_ exposure accumulation over time, it is recommended that various air pollution controls be put in place, such as the installation of a vented range hood located above the stove or cooking apparatus for those inside stalls, a total market air pollution-control programme, controls on vehicle emissions, and the restructuring of vending sites or markets, such as having markets not very close to the roads and industrial or offensive trades. Furthermore, vendors should be placed in sustainable education programs that include air pollution and the importance of respiratory protective equipment usage. Furthermore, vendors should use cleaner cooking fuels.

## Figures and Tables

**Table 1:** Cross-tabulation of personal sampling PM_2.5_ concentrations and homogeneous exposure groups (HEG).

PM_2.5_ TWA Concentrations (mg/m^3^)		Homogeneous Exposure Group (HEG)			Total n (%)
		HEG-1 (n)	>HEG-2 (n)	>HEG-3 (n)	
	<0.01	3	1	1	5 (20%)
	0.01	2	1	1	4 (16%)
	0.02	1	0	0	1 (4%)
	0.04	1	0	0	1 (4%)
	0.09	1	0	0	1 (4%)
	0.11	0	2	0	2 (8%)
	0.12	0	0	1	1 (4%)
	0.16	0	2	1	3 (12%)
	0.17	0	1	0	1 (4%)
	0.18	0	1	0	1 (4%)
	0.24	1	0	0	1 (4%)
	0.41	0	1	0	1 (4%)
	0.48	0	1	0	1 (4%)
	0.62	0	0	1	1 (4%)
	0.77	1	0	0	1 (4%)
**Total number of samples N (25)**		**10 (40%)**	**10 (40%)**	**5 (20%)**	**25 (100%)**

**Table 2:** Descriptive statistics of TWA PM_2.5_ concentrations of informal cooking vendors in the inner city of Johannesburg.

Air Pollutant	Area of Monitoring	Mean	Range	South African Standards	No. of Samples Exceeding Limit	
					Indoor	Outdoor
8 h-PM_2.5_ (mg/m^3^)	Indoor Makert (HEG-1)	0.12	<0.01–0.77	5 mg/m^3^	0	0
8 h-PM_2.5_ (mg/m^3^)	Outoor Makert (HEG-2)	0.18	<0.01–0.48	5 mg/m^3^	0	0
8 h-PM_2.5_ (mg/m^3^)	Outdoor Makert (HEG-3)	0.18	<0.01–0.62	5 mg/m^3^	0	0

## Data Availability

All data in this study were provided in the main manuscript.

## Supplementary Materials

The following supporting information can be downloaded at: https://www.mdpi.com/article/10.3390/ijerph20032465/s1, Table S1: Homogeneous exposure grouping for the Johannesburg inner city markets; Table S2: PM_2.5_ TWA concentrations amongst informal cooking vendors in the inner city, Johannesburg (June–August 2022).
