# Supplementary material for: Personal PM2.5 Exposure Monitoring of Informal Cooking Vendors at Indoor and Outdoor Markets in Johannesburg, South Africa
Source: Int J Environ Res Public Health. 2023 Jan 30;20(3):2465. doi: 10.3390/ijerph20032465 (PMC9915915; doi:10.3390/ijerph20032465)
Supplement: Supplementary file 1 [file ijerph-20-02465-s001.zip › ijerph-2149129-supplementary.pdf]

Table S1: Homogeneous exposure grouping for the Johannesburg inner city Markets

MARKET NO.	WORK LOCATION	SITE OF MAKERT	TYPE OF SHELTER	OPERATING/STALL AREA FLOOR	OPERATING/STALL AREA WALL	OPERATING/STALLS ROOF	COOKING HEAT OR FUELS USED	MARKET SURROUNDING SCENERY	HOMOGENEOUS EXPOSURE GROUP
									**HEG 1=** Indoor/inside building market (use electricity and gas)
									**HEG 2=** Outdoor (use open fire/charcoal).
									**HEG 3=** Outdoor (road side/Sidewalk/roadside) market (use gas)
1.	Indoor	Transportation hub (Taxi rank)	Full enclosed	Cemented	Brick walls	Metal roof	Electricity and gas	Transportation within the building, (outside building- residential flats,offices , shops and highway and local traffic)	**HEG 1**
2.	Outdoor	Sidewalk/roadside	Partially enclosed	Street pavement	Half brick wall	Steel	Gas stoves	Residential Flats, transport stations, office building, shops, offices, traffic	**HEG 3**
3.	Outdoor	Fenced site	Open operational areas (enclosed storages)	Paved	No wall for cooking area Steel sides	no cover for cooking areas and steel steel for storage stall	Open fire, and gas	Residential Flats, transport stations, office building, shops, offices, traffic,	**HEG 2**

							industrial premise, offensive trades	
4.	Indoor	Market building	Full enclosed	Cemented	Brick walls	Metal roof	Electricity and gas	No visual inside(outside building- residential flats,offices , shops and traffic)	**HEG 1**
5.	Outdoor	Sidewalk/roadside market	Only roof cover	Street pavement	None	Top cover steel shelter	None	Outside	**HEG 3**
6.	Indoor	Market building	Fully enclosed	Cemented	Brick walls	Metal roof	Electricity and gas	No visual inside(outside building- residential flats,offices , shops and traffic)	**HEG 1**
7.	Indoor	Market building	Fully enclosed	Cemented	Brick walls	Metal roof	Electricity and gas	No visual inside(outside building- residential flats,offices , shops and traffic)	**HEG 1**
8.	Outdoor	Sidewalk/roadside market	Only roof cover	Street pavement	Cloth, cardbox	Top cover steel shelter	Gas stove	Residential Flats, transport stations, office building, shops, offices, traffic	**HEG 3**
9.	Outdoor	Sidewalk/roadside market	Only roof cover	Street pavement	Tent, card box	Top cover steel shelter	Gas stove	Residential Flats, transport stations, office building, shops, offices, traffic,	**HEG 3**
10.	Outdoor	Sidewalk/roadside	No shelter	Street pavement	Cloth,	No shelter	Gas stove/few open fires	Residential Flats, transport stations, office building, shops, offices, traffic	**HEG 2**
11.	Outdoor	Sidewalk/roadside	No shelter	Street pavement	Tent, card box	No shelter	Gas stove/few open fires	Residential Flats, transport stations, office building, shops, offices, traffic	**HEG 2**

12.	Indoor	Transportation hub (Railway, bus and taxi rank)	Fully enclosed	Street pavement	Cloth,	Metal roof	Electricity and gas	Transportation within the building, (outside building- residential flats,offices , shops and traffic)	**HEG 1**
13.	Indoor	Makert building	Full enclosed cooking vendor and roof closure for fruits and vegetable stalls	Cemented	Tent, card box	Metal roof	Electricity/gas, few open fires	outside building- residential flats,offices , shops and traffic	**HEG 1**
14.	Outdoor	Sidewalk/roadside	No shelter	Street pavement	Cloth,	No shelter	Gas stove/ few open fires stall	(Residential Flats, transport stations, office building, shops, offices, traffic	**HEG 2**
15.	Indoor	Transportation hub (Taxi rank)	Fully enclosed	Cemented	Tent, card box	Metal roof	Electricity/gas	Transportation within the building, (outside building- residential flats,offices , shops and traffic	**HEG 1**
16.	Outdoor	Transportation hub (Railway station)	Open operational areas and enclosed storages)	Sandy/soil in most stall operating areas (Cemented in storages)	Brick walls	Metal roof	Gas and open fire	Residential Flats, transport stations, office building, shops, offices, traffic	**HEG 2**

Table S2. PM2.5 TWA concentrations amongst informal cooking vendors in the inner city, Johannesburg (June-august 2022).

Sampling date	Sample No.	Worker identification No	Occupation	Sampling Area	Sample Duration (Min)	Substance	TWA Concentration (mg/m^3)		Risk Rating	Evaluation of Results and Findings
							Measured	OEL		
**VENDOR MAKERT 1 (TOTAL OF 10 SAMPLES)**										
**HOMOGENEOUS EXPOSURE GROUP 1: INDOOR VENDOR MARKET WITH ELECTRICAL AND GAS STOVES ACTIVITIES**										
**TOTAL STALLs POPULATION: 25**										
30th June 2022	PVC/11/73/2022	HEG-1-01	**Indoor cooking vendor**	**Vendors Market 1-HEG-1**	371	$PM_{2.5}$	<0.01	5.0		$PM_{2.5}$ below occupational exposure limits.
30th June 2022	PVC/11/69/2022	HEG-1-02	Indoor cooking vendor	**Vendors Market 1-HEG-1**	366	$PM_{2.5}$	<0.01	5.0		$PM_{2.5}$ below occupational exposure limits.
30th June 2022	PVC/11/71/2022	HEG-1-03	Indoor cooking vendor	**Vendors Market 1-HEG-1**	366	$PM_{2.5}$	0.01	5.0		$PM_{2.5}$ below occupational exposure limits.
30th June 2022	PVC/11/72/2022	HEG-1-04	Indoor cooking vendor	**Vendors Market 1-HEG-1**	369	$PM_{2.5}$	<0.01	5.0		$PM_{2.5}$ below occupational exposure limits.

30th June 2022	PVC/10/72/2022	HEG-1-05	Indoor cooking vendor	**Vendors Market 1-HEG-1**	367	$PM_{2.5}$	0.04	5.0		$PM_{2.5}$ below occupational exposure limits.
24th August 2022	MCE/4/12/2022	HEG-1-06	Indoor cooking vendor	**Vendors Market 1-HEG-1**	370	$PM_{2.5}$	0.09	5.0		$PM_{2.5}$ below occupational exposure limits.
24th August 2022	MCE4/1/2022	HEG-1-07	Indoor cooking vendor	**Vendors Market 1-HEG-1**	363	$PM_{2.5}$	0.02	5.0		$PM_{2.5}$ below occupational exposure limits.
24th August 2022	MCE/4/17/2022	HEG-1-08	Indoor cooking vendor	**Vendors Market 1-HEG-1**	362	$PM_{2.5}$	0.77	5.0		$PM_{2.5}$ below occupational exposure limits.
24th August 2022	MCE/4/2/2022	HEG-1-09	Indoor cooking vendor	**Vendors Market 1-HEG-1**	363	$PM_{2.5}$	0.01	5.0		$PM_{2.5}$ below occupational exposure limits.
24th August 2022	MCE/4/19/2022	HEG-1-10	Indoor cooking vendor	**Vendors Market 1-HEG-1**	360	$PM_{2.5}$	0.24	5.0		$PM_{2.5}$ below occupational exposure limits.
**VENDEOR MAKERT 3 (TOTAL OF 10 SAMPLES)**										
**HOMOGENEOUS GROUP 2: OUTDOOR MARKET WITH OPEN FIRE ACTIVITIES**										
**TOTAL VENDOR STALLS POPULATION: 30**										
24th August 2022	MCE/4/17/2022	HEG-2-01	Outdoor cooking vendor	**Vendors Market 3-HEG-2**	364	$PM_{2.5}$	0.01	5.0		$PM_{2.5}$ below occupational exposure limits.

24th August 2022	MCE/4/15/2022	HEG-2-02	Outdoor cooking vendor	**Vendors Market 3-HEG-2**	363	$PM_{2.5}$	0.16	5.0		$PM_{2.5}$ below occupational exposure limits.
24th August 2022	MCE/4/16/2022	HEG-2-03	Outdoor cooking vendor	**Vendors Market 3-HEG-2**	358	$PM_{2.5}$	0.48	5.0		$PM_{2.5}$ below occupational exposure limits.
24th August 2022	MCE/4/5/2022	HEG-2-04	Outdoor cooking vendor	**Vendors Market 3-HEG-2**	362	$PM_{2.5}$	<0.01	5.0		$PM_{2.5}$ below occupational exposure limits.
24th August 2022	MCE/4/4/2022	HEG-2-05	Outdoor cooking vendor	**Vendors Market 3-HEG-2**	363	$PM_{2.5}$	0.11	5.0		$PM_{2.5}$ below occupational exposure limits.
24th August 2022	MCE/4/14/2022	HEG-2-06	Outdoor cooking vendor	**Vendors Market 3-HEG-2**	363	$PM_{2.5}$	0.18	5.0		$PM_{2.5}$ below occupational exposure limits.
24th August 2022	MCE/4/8/2022	HEG-2-07	Outdoor cooking vendor	**Vendors Market 3-HEG-2**	361	$PM_{2.5}$	0.11	5.0		$PM_{2.5}$ below occupational exposure limits.
24th August 2022	MCE/4/13/2022	HEG-2-08	Outdoor cooking vendor	**Vendors Market 3-HEG-2**	360	$PM_{2.5}$	0.41	5.0		$PM_{2.5}$ below occupational exposure limits.
24th August 2022	MCE/4/7/2022	HEG-2-09	Outdoor cooking vendor	**Vendors Market 3-HEG-2**	361	$PM_{2.5}$	0.17	5.0		$PM_{2.5}$ below occupational exposure limits.
24th August 2022	MCE/4/18/2022	HEG-2-10	Outdoor cooking vendor	**Vendors Market 3-HEG-2**	360	$PM_{2.5}$	0.16	5.0		$PM_{2.5}$ below occupational exposure limits..

VENDOR MARKET 2 (TOTAL OF 5 SAMPLES)

**HOMOGENEOUS EXPOSURE GROUP 3: OUTDOOR MARKET WITH GAS STOVE ACTIVITIES (EXPOSED DIRECTLY TO TRANSPORTATION AND CONSTRUCTION)**									
**TOTAL STALLS POPULATION: 9**									
30th June 2022	PVC/11/74/2022	01	Street Vendor	**Vendors Market 2-HEG-3**	361	$PM_{2.5}$	0.01	5.0		$PM_{2.5}$ below occupational exposure limits.
30th June 2022	PVC/11/68/2022	02	Street Vendor	**Vendors Market 2-HEG-3**	363	$PM_{2.5}$	<0.01	5.0		$PM_{2.5}$ below occupational exposure limits.
30th June 2022	PVC/11/65/2022	03	Street Vendor	**Vendors Market 2-HEG-3**	362	$PM_{2.5}$	0.12	5.0		$PM_{2.5}$ below occupational exposure limits.
30th June 2022	PVC/11/67/2022	04	Street Vendor	**Vendors Market 2-HEG-3**	360	$PM_{2.5}$	0.16	5.0		$PM_{2.5}$ below occupational exposure limits..
30th June 2022	PVC/11/66/2022	05	Street Vendor	**Vendors Market 2-HEG-3**	363	$PM_{2.5}$	0.62	5.0		$PM_{2.5}$ below occupational exposure limits
